# Fully fledged enantiornithine hatchling revealed by Laser-Stimulated Fluorescence supports precocial nesting behavior

**DOI:** 10.1038/s41598-019-41423-7

**Published:** 2019-03-21

**Authors:** Thomas G. Kaye, Michael Pittman, Jesús Marugán-Lobón, Hugo Martín-Abad, José Luis Sanz, Angela D. Buscalioni

**Affiliations:** 1 Foundation for Scientific Advancement, Sierra Vista, Arizona 85650 United States of America; 2 Vertebrate Palaeontology Laboratory, Department of Earth Sciences, The University of Hong Kong, Pokfulam, Hong Kong SAR China; 3 Facultad de Ciencias, Departamento de Biología, Universidad Autónoma de Madrid, 28049 Madrid, Spain

## Abstract

Laser-Stimulated Fluorescence (LSF) is used to identify fully fledged feathering in the hatchling enantiornithine bird specimen MPCM-LH-26189, supporting precocial nesting behavior in this extinct group. The LSF results include the detection of a long pennaceous wing feather as well as cover feathers around the body. The LSF technique showed improved detection limits over and above synchrotron and UV imaging which had both been performed on this specimen. The findings underscore the value of using a wide range of analytical techniques.

## Introduction

**Figure 1:**
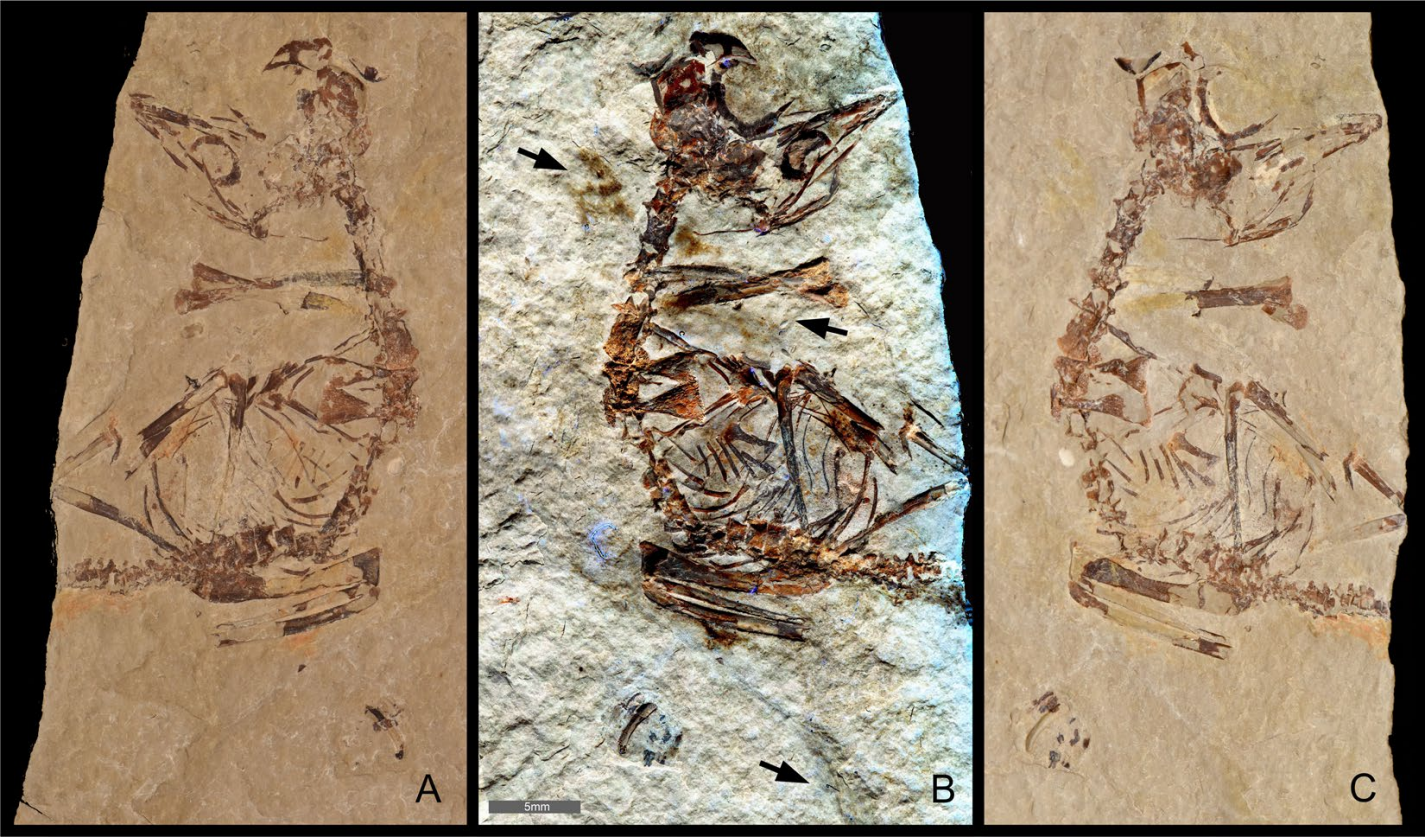
Spanish enantiornithine hatchling MPCM-LH-26189. (**A**) White light image of the counterslab. (**B**) Laser-Stimulated Fluorescence (LSF) image of the slab and counterslab combined (composite image) reveals brown patches around the specimen. These comprise of clumps of elongate feathers associated with the neck and wings (upper arrows; see Figs 2 and 3 for close-up images) as well as a single long pennaceous feather associated with the left wing (lower arrow; see Fig. 2E,F for close-up image). (**C**) White light image of the slab. Scale = 5 mm.

The enantiornithine hatchling MPCM-LH-26189 from the Las Hoyas locality of Spain helped to identify an asynchronous clade-wide pattern of sternal and vertebral osteogenesis in early juvenile enantiornithines, supporting variation in their size and their tempo of skeletal maturation^1^. This previous study found no feathers or chemical evidence for plumage (see Fig. 5 caption of^1^) with faint ribbing visible in a yellowish stain suggested to be more consistent with the morphology of vegetal material than with feathers (see Supplementary Note 1 of^1^). MPCM-LH-26189 is reasonably well articulated and has some soft-tissue-associated chemistry^1^. These lines of evidence were used to suggest that MPCM-LH-26189 might have been largely featherless when it died (see Supplementary Note 1, Supplementary Figs 2–5 and Supplementary Table 2 of^1^). While unconfirmed (see Fig. 5 caption of^1^) this implies a developmental strategy towards the altricial end of the altricial-precocial developmental spectrum, which would be unexpected evidence of enantiornithine reproductive behavior. Given the exceptional preservation of the Las Hoyas fossil record^2^, and of feathers in particular^3,4^, here we evaluate the developmental strategy of MPCM-LH-26189 using Laser-Stimulated-Fluorescence (LSF)^5^ to identify possible additional evidence of preserved feathers. For instance, in MPCM-LH-26189 yellowish stains are preserved in various positions across the entire body (Fig. 1), suggesting they were remnants of the entire body-contour. LSF does not identify specific elemental signatures, but it does differentially fluoresce extremely small differences in mineral lattice contamination detectable as color differences. It has also been successfully employed in the study of other Las Hoyas fossils^6^. Detectable fluorescence at the parts per million level is not uncommon^5,7^. Further details of the technique are provided in the Methods section.

## Results

**Figure 2:**
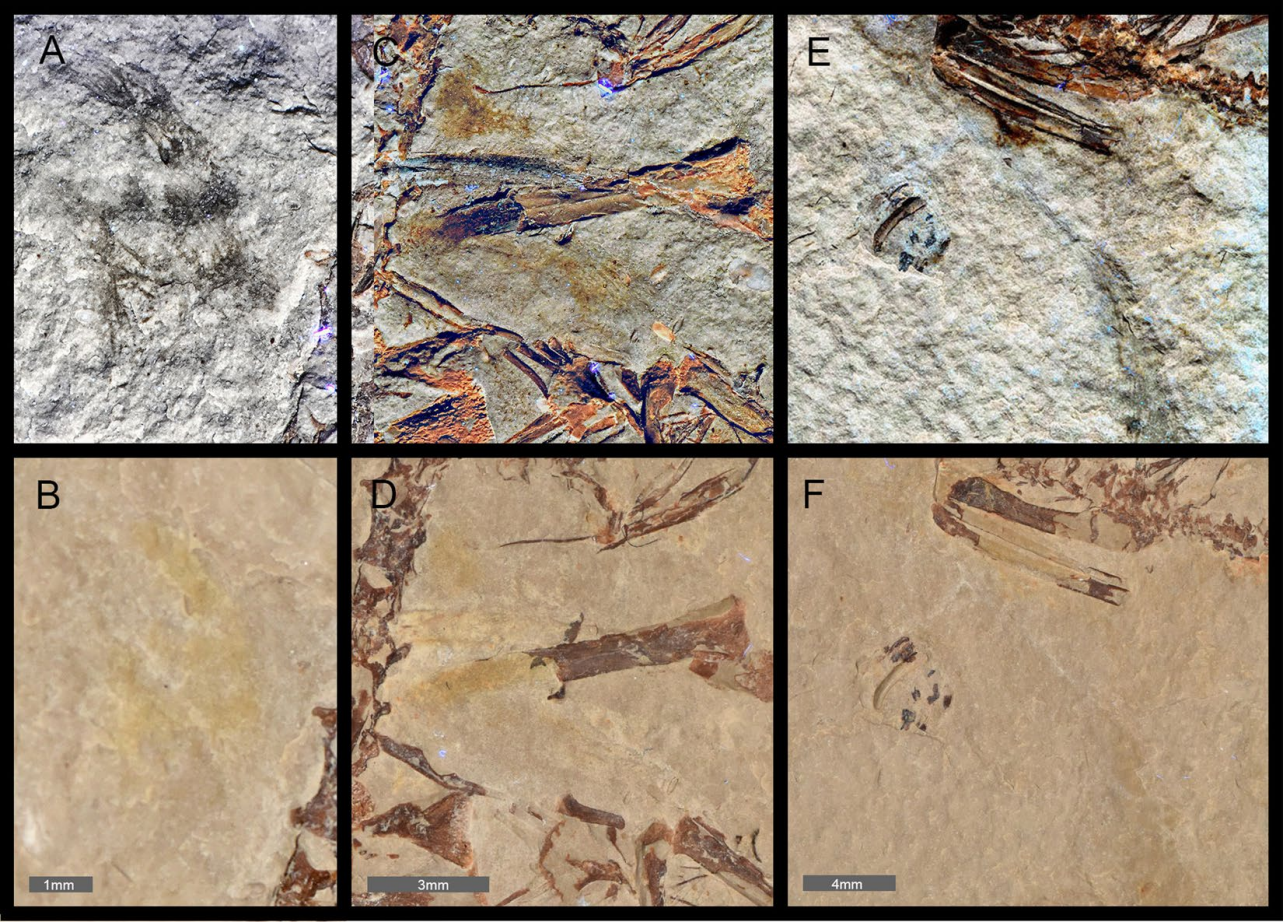
Preserved feathering of Spanish enantiornithine hatchling MPCM-LH-26189 under LSF and white light. Elongate feathers preserving bushy dorsal tips are found near the neck and appear to be cover feathers: (**A**) under LSF, (**B**) under white light. Scale = 1 mm. Suspected feather clumps are associated with the right wing (**C**) under LSF, (**D**) under white light. Scale = 3 mm. A long pennaceous feather associated with the left wing is very similar to an enantiornithine embryo specimen from China (**E**) under LSF, (**F**) under white light. Scale = 4 mm.

**Figure 3:**
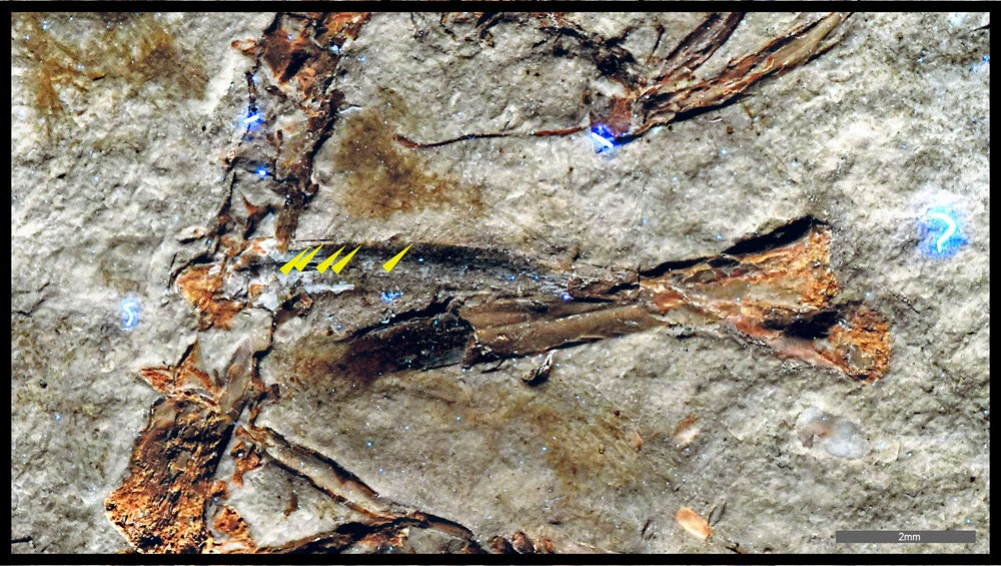
Right wing of the Spanish enantiornithine hatchling MPCM-LH-26189. Filaments preserved adjacent to the ulna (yellow arrows) as seen in the combined LSF images of the slab and counterslab (composite image). Scale = 2 mm.

The LSF scans of the yellowish stains, such as near the neck and wings, showed brown-colored patches containing filament structures (Figs 1–3). This indicates that the Las Hoyas hatchling did in fact have feathers. This is consistent with findings in early stage enantiornithines^8–12^. These areas were also recovered as colored patches or chemical ‘ghosts’ in previous UV images and SRS-XRF elemental maps (see Figs S2, S4 and Supplementary Note 1 of^1^), but their structural details were not apparent using those techniques. Inspection of the matrix (Fig. 1A,C) rules out preparation marks as a possible origin of these structures. Furthermore, the LSF images are consistent with the morphology of the Las Hoyas specimens *Eoalulavis* MPCM-LH-13500^13,14^, *Concornis* MPCM-LH-2814^13,15,16^ and isolated feathers^4^.

The brown-colored feather patches appear across the entire body (Fig. 1B) and show the best detail on the left side of the neck and next to the left wing. The patch on the left side of the neck (Figs 1 and 2A,B) comprises elongate structures banded with lighter and darker color, with a ‘bushy’ appearance. In the right wing, there are two patches associated with the ulna and radius (Figs 2C and 3). Interestingly, multiple thin filaments stem obliquely from the ulna shaft (Figs 2C and 3). However, the most noteworthy feather is associated with the left wing and is anatomically displaced, close to the sacrum (Figs 1B and 2E). This feather is ~3 cm long and barbs are well-preserved along different portions of the hollow rachis with those at the distal end forming an angle of ~30°. This wing feather is very similar in both anatomy, location and relative size to an enantiornithine embryo specimen from China^9^.

## Discussion

The preservation of feathers in MPCM-LH-26189, as revealed by LSF, indicates that this hatchling was fully fledged and not largely featherless. This supports precociality as a nesting behavior in enantiornithines. It is noteworthy that the long wing feather of MPCM-LH-26189 is extremely similar to the Chinese enantiornithine embryo specimen that was first used to propose precociality in this group^9^. MPCM-LH-26189 is highly articulated, which is congruent with the body fossil and soft tissue preservation in the enantiornithines of Las Hoyas^3^. According to the LSF results, there are two types of feathers in MPCM-LH-26189, raising the question of whether this confirms both remigial and cover feathers. Such an assertion is important because cover feathers have never been documented in enantiornithines. In this specimen, some preserved feathers are located near the neck (Fig. 2A), a body region of modern birds that is only known to have cover feathers^8^. The fact that the specimen is an unambiguous hatchling means that such cover feathers would be the enantiornithine-equivalent of down feathers in crown birds. If so, this first evidence could serve as a clue to identify adult enantiornithine cover feathers, which remain elusive.

Synchrotron generated x-ray beams were applied to MPCM-LH-26189^1^, which has become a popular method of analyzing fossils^17–21^. Due to micron scale beam size they are exquisite at resolving the smallest details and fine scale differences in matrix-fossil density^18^. Combined with Energy Dispersive Spectroscopy, they are capable of detailed and sensitive spectral analysis resulting in previously unseen elemental maps of soft tissue residues^20^. However, synchrotron hutches are typically open air or employ helium tents because of their inherent lack of sensitivity to elements at the lower end of the atomic scale^22^. Unless operating in a high vacuum, lower energy x-rays are absorbed by the intervening gas^23^. Silicon is a typical cut off point, so carbon and other lighter elements would not normally be in the detectable range^23^, potentially overlooking morphologies that they preserve. Typically, fossil feathers are easily visible in white light as carbon films^24^. Carbon is a very low fluorescence element^25^, so under LSF, feathers are typically black and show up in high contrast by the fluorescence of the background matrix^5,7,26,27^. However, the Las Hoyas hatchling showed no evidence of carbon films under white light or in previous UV and synchrotron imaging^1^ (Fig. 1A; Fig. 1 of^1^). This suggests the possibility that there is too low a percentage of carbon to be easily visible in the matrix, but under LSF, the residual carbon (or possibly the detected iron^1^) quenches the fluorescence^25^ in the local area revealing the feather filaments.

In bridging detection limits in synchrotron and UV analytical techniques, Laser-Stimulated Fluorescence (LSF), a rapid, low-cost technique^5^, has helped to clarify the developmental strategy of MPCM-LH-26189 and of enantiornithines more generally. This example underscores the range of data that remains undiscovered in important fossils, and the value of adopting a broader analytical repertoire.

## Methods

Laser-Stimulated Fluorescence (LSF) imaging followed the protocol of Kaye *et al*.^5^, which was developed in^7,26,28,29^. Thus, only an abbreviated version is provided here. A 405 nm laser diode was used to fluoresce the specimen follow standard laser safety protocol. 30 second time-exposed images were taken with a Nikon D810 DSLR camera and a 425 nm laser blocking filter. Post processing applied uniformly across entire images (equalization, saturation and color balance) was performed in Photoshop CS6 software.

## References

[1] Knoll F (2018). A diminutive perinate European Enantiornithes reveals an asynchronous ossification pattern in early birds. Nature Communications.

[2] Poyato-Ariza, F. J. & Buscalioni, A. D. *Las Hoyas: a Cretaceous wetland: a multidisciplinary synthesis after 25 years of research on an exceptional fossil Lagerstatte from Spain*. (Pfeil Verlag, 2016).

[3] Navalón G, Marugán-Lobón J, Chiappe LM, Sanz JL, Buscalioni ÁD (2015). Soft-tissue and dermal arrangement in the wing of an Early Cretaceous bird: Implications for the evolution of avian flight. Scientific Reports.

[4] Marugán-Lobón J, Vullo R (2011). Feather diversity in the Barremian (Early Cretaceous) of Las Hoyas, Spain. Comptes Rendus Palevol.

[5] Kaye TG (2015). Laser-stimulated fluorescence in paleontology. PLOS ONE.

[6] Poyato-Ariza, F. J. *et al*. In *8th International Meeting on Taphonomy and Fossilization* (Vienna, Austria, 2017).

[7] Wang XL (2017). Basal paravian functional anatomy illuminated by high-detail body outline. Nature Communications.

[8] Lucas, A. M. & Stettenheim, P. R. *Avian anatomy: integument*. (US Department of Agriculture, 1972).

[9] Zhou ZH, Zhang FC (2004). A precocial avian embryo from the Lower Cretaceous of China. Science.

[10] Xing LD (2018). A flattened enantiornithine in mid-Cretaceous Burmese amber: morphology and preservation. Science Bulletin.

[11] Xing LD (2017). A mid-Cretaceous enantiornithine (Aves) hatchling preserved in Burmese amber with unusual plumage. Gondwana Research.

[12] Xing LD (2016). Mummified precocial bird wings in mid-Cretaceous Burmese amber. Nature Communications.

[13] Sanz JL, Ortega F (2002). The birds from Las Hoyas. Science Progress.

[14] Sanz JL, Chiappe LM, Perez-Moreno BP, Buscalioni AD, Moratalla JJ (1996). A new Lower Cretaceous bird from Spain: implications for the evolution of flight. Nature.

[15] Sanz JL, Chiappe LM, Buscalioni AD (1995). The osteology of *Concornis lacustris* (Aves: Enantiornithes) from the Lower Cretaceous of Spain and a reexamination of its phylogenetic relationships. American Museum Novitates.

[16] Sanz JL, Buscalioni AD (1992). A new bird from the early Cretaceous of Las Hoyas, Spain, and the early radiation of birds. Palaeontology.

[17] Bergmann, U. *et al*. *Archaeopteryx* feathers and bone chemistry fully revealed via synchrotron imaging. *Proceedings of the National Academy of Sciences* **107**, 9060–9065 (2010). 10.1073/pnas.1001569107 PMC2889062 20457935

[18] Cau A (2017). Synchrotron scanning reveals amphibious ecomorphology in a new clade of birdlike dinosaurs. Nature.

[19] Manning PL (2013). Synchrotron-based chemical imaging reveals plumage patterns in a 150 million year old early bird. Journal of Analytical Atomic Spectrometry.

[20] Bergmann U, Manning PL, Wogelius RA (2012). Chemical mapping of paleontological and archeological artifacts with synchrotron x-rays. Annual Review of Analytical Chemistry.

[21] Sanchez S, Ahlberg PE, Trinajstic K, Mirone A, Tafforeau P (2012). Three-dimensional synchrotron virtual paleohistology: a new insight into the world of fossil bone microstructures. Microscopy and Microanalysis.

[22] Lytle, F. W. In *Applica*tions *o*f syn*chrotron radiation* (eds Winick, H., Xian, D., Ye, M. H. & Huang, T.) (Gordon & Breach, 1989).

[23] Brown, G. E. Jr. & Waychunas, G. A. *X-ray absorption spectroscopy: introduction to experimental procedures*, 2004.

[24] Davis PG, Briggs DEG (1995). Fossilization of feathers. Geology.

[25] Kagan MR, McCreery RL (1994). Reduction of fluorescence interference in Raman spectroscopy via analyte adsorption on graphitic carbon. Analytical Chemistry.

[26] Kaye TG, Pittman M, Mayr G, Schwarz D, Xu X (2019). Detection of lost calamus challenges identity of isolated *Archaeopteryx* feather. Scientific Reports.

[27] Falk AR, Kaye TG, Zhou ZH, Burnham DA (2016). Laser fluorescence illuminates the soft tissue and life habits of the Early Cretaceous bird *Confuciusornis*. PLOS ONE.

[28] Xu X (2017). Modular evolution in an asymmetrically feathered troodontid with transitional features. Nature Communications.

[29] Yang ZX (2018). Pterosaur integumentary structures with complex feather-like branching. *Nature Ecology &*. Evolution.

